# Supplementary material for: Impaired Generation of Transit-Amplifying Progenitors in the Adult Subventricular Zone of Cyclin D2 Knockout Mice
Source: Cells. 2022 Jan 1;11(1):135. doi: 10.3390/cells11010135 (PMC8750346; doi:10.3390/cells11010135)
Supplement: Supplementary file 1 [file cells-11-00135-s001.zip › cells-1467224_Suppl. Table S1_Phenotypes as % of Hoechst.pdf]

Mice	Section	No. of cells Hoechst(+) taken as 100%	B1q (EdU-)		mean [%]	B1a (EdU+/-)		mean [%]	B1a (EdU+)		mean [%]	TaPs (EdU+/-)		mean [%]	TaPs (EdU+)		mean [%]	Others (EdU+/-)		mean [%]
			**No.**	**%**		**No.**	**%**		**No.**	**%**		**No.**	**%**		**No.**	**%**		**No.**	**%**	
	1	1417	191	13.5		98	6.9		12	0.8		191	13.5		34	2.4		937	66.1	
WT_#1	2	1500	140	9.3	**10.7**	73	4.9	**5.2**	10	0.7	**0.6**	264	17.6	**14.5**	32	2.1	**2.2**	1023	68.2	**69.6**
	3	1633	150	9.2		62	3.8		6	0.4		205	12.6		35	2.1		1216	74.5	
	1	1840	379	20.6		326	17.7		50	2.7		312	17.0		57	3.1		823	44.7	
WT_#2	2	1361	139	10.2	**14.9**	139	10.2	**10.5**	23	1.7	**1.6**	284	20.9	**16.8**	26	1.9	**2.4**	799	58.7	**57.9**
	3	1364	188	13.8		48	3.5		4	0.3		170	12.5		30	2.2		958	70.2	
	1	2010	298	14.8		171	8.5		29	1.4		309	15.4		45	2.2		1232	61.3	
WT_#3	2	1814	245	13.5	**16.3**	198	10.9	**8.3**	25	1.4	**1.1**	433	23.9	**18.0**	102	5.6	**3.5**	938	51.7	**57.4**
	3	2118	434	20.5		114	5.4		11	0.5		314	14.8		55	2.6		1256	59.3	
**mean [%]** (± SD)					**13.9** (± 2.9**)**			**8.0** (± 2.7)			**1.1** (± 0.5)			**16.4** (± 1.8)			**2.7** (± 0.7)			**61.6** (± 6.9)
	1	1013	181	17.9		131	12.9		7	0.7		119	11.7		3	0.3		582	57.5	
KO_#1	2	1082	149	13.8	**16.2**	65	6.0	**7.7**	5	0.5	**0.4**	66	6.1	**7.0**	2	0.2	**0.2**	802	74.1	**69.0**
	3	1100	187	17.0		47	4.3		2	0.2		36	3.3		1	0.1		830	75.5	
	1	976	156	16.0		66	6.8		0	0.0		86	8.8		2	0.2		668	68.4	
KO_#2	2	1151	165	14.3	**15.3**	74	6.4	**6.1**	1	0.1	**0.1**	69	6.0	**6.5**	7	0.6	**0.3**	843	73.2	**72.1**
	3	1069	165	15.4		55	5.1		2	0.2		50	4.7		0	0.0		799	74.7	
	1	910	188	20.7		99	10.9		4	0.4		45	4.9		0	0.0		578	63.5	
KO_#3	2	917	123	13.4	**15.4**	86	9.4	**8.3**	4	0.4	**0.4**	38	4.1	**4.5**	0	0.0	**0.0**	670	73.1	**71.9**
	3	1270	153	12.0		59	4.6		3	0.2		55	4.3		0	0.0		1003	79.0	
**mean [%]** (± SD)					**15.6** (± 0.5)			**7.4** (± 1.1)			**0.3** (± 0.2)			**6.0** (± 1.4)			**0.2** (± 0.1)			**71.0** (± 1.7)
**P value** (WT vs KO)					P = 0.4249			P = 0.7457			P = 0.0839			P = 0.0016 **			P = 0.0197 *			P = 0.1359
