# Supplementary material for: Impaired Generation of Transit-Amplifying Progenitors in the Adult Subventricular Zone of Cyclin D2 Knockout Mice
Source: Cells. 2022 Jan 1;11(1):135. doi: 10.3390/cells11010135 (PMC8750346; doi:10.3390/cells11010135)
Supplement: Supplementary file 1 [file cells-11-00135-s001.zip › cells-1467224_Suppl. Table S2_EdU(+) B1a and TaPs as % of their populations.pdf]

**Mice**	**Section**	**No. of B1a (EdU+/-) taken as 100%**	**B1a** (EdU+)		mean [%]	**No. of TaPs (EdU+/-) taken as 100%**	**TaPs** (EdU+)		mean [%]
			**No.**	**%**			**No.**	**%**	
WT_#1	1	98	12	12.2	**11.9**	191	34	17.8	**15.7**
	2	73	10	13.7		264	32	12.1	
	3	62	6	9.7		205	35	17.1	
WT_#2	1	326	50	15.3	**13.4**	312	57	18.3	**15.0**
	2	139	23	16.5		284	26	9.2	
	3	48	4	8.3		170	30	17.6	
WT_#3	1	171	29	17.0	**13.1**	309	45	14.6	**18.5**
	2	198	25	12.6		433	102	23.6	
	3	114	11	9.6		314	55	17.5	
**mean [%]** (± SD)					**12.8** (± 0.8)				**16.4** (± 1.9)
KO_#1	1	131	7	5.3	**5.8**	119	3	2.5	**2.8**
	2	65	5	7.7		66	2	3.0	
	3	47	2	4.3		36	1	2.8	
KO_#2	1	66	0	0.0	**1.7**	86	2	2.3	**4.2**
	2	74	1	1.4		69	7	10.1	
	3	55	2	3.6		50	0	0.0	
KO_#3	1	99	4	4.0	**4.6**	45	0	0.0	**0.0**
	2	86	4	4.7		38	0	0.0	
	3	59	3	5.1		55	0	0.0	
**mean [%]** (± SD)					**4.0** (± 2.1)				**2.3** (± 2.1)
**P value** (WT vs KO)					P = 0.0107 *				P = 0.0010 **
