# Supplementary figures and images for: Impaired Generation of Transit-Amplifying Progenitors in the Adult Subventricular Zone of Cyclin D2 Knockout Mice
Source: Cells. 2022 Jan 1;11(1):135. doi: 10.3390/cells11010135 (PMC8750346; doi:10.3390/cells11010135)

WT

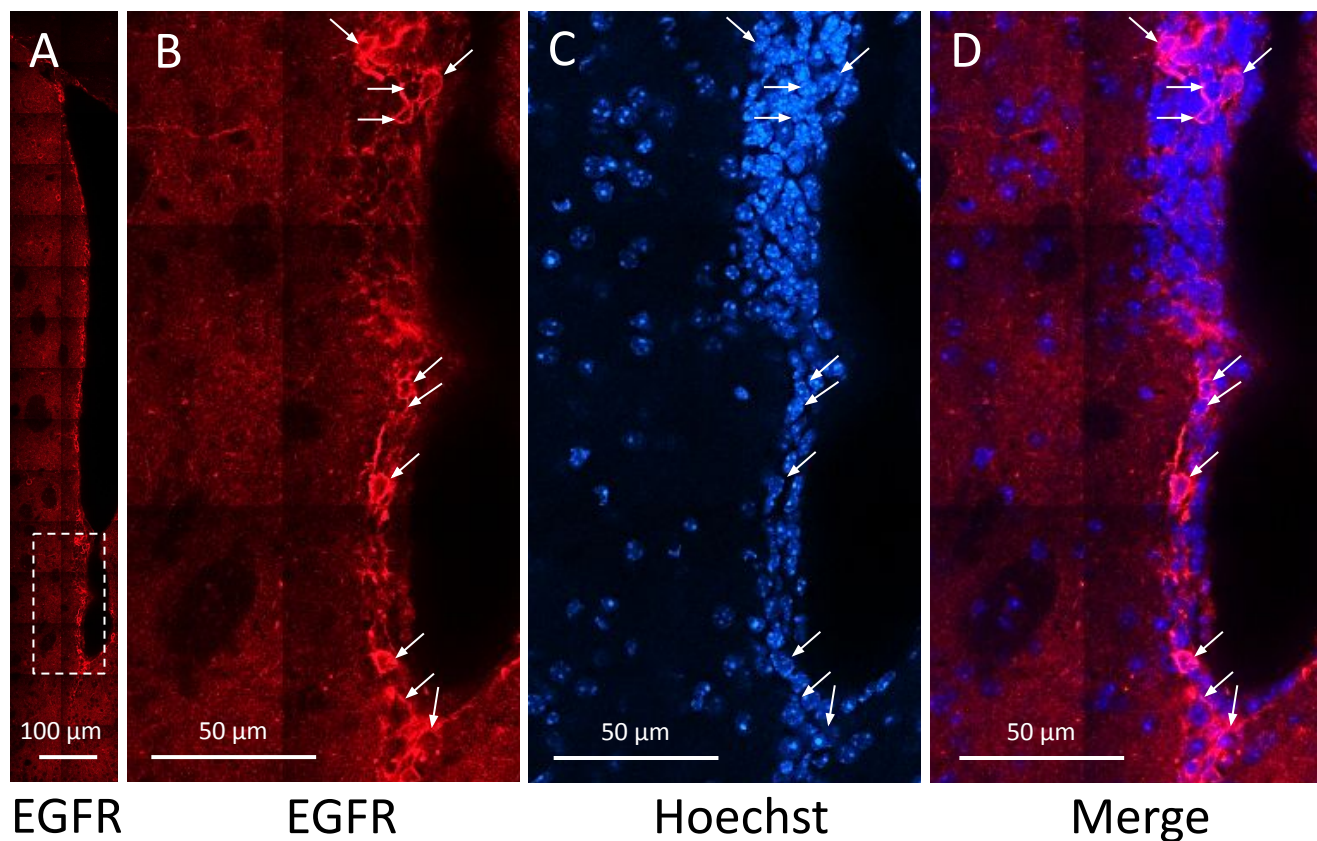

KO

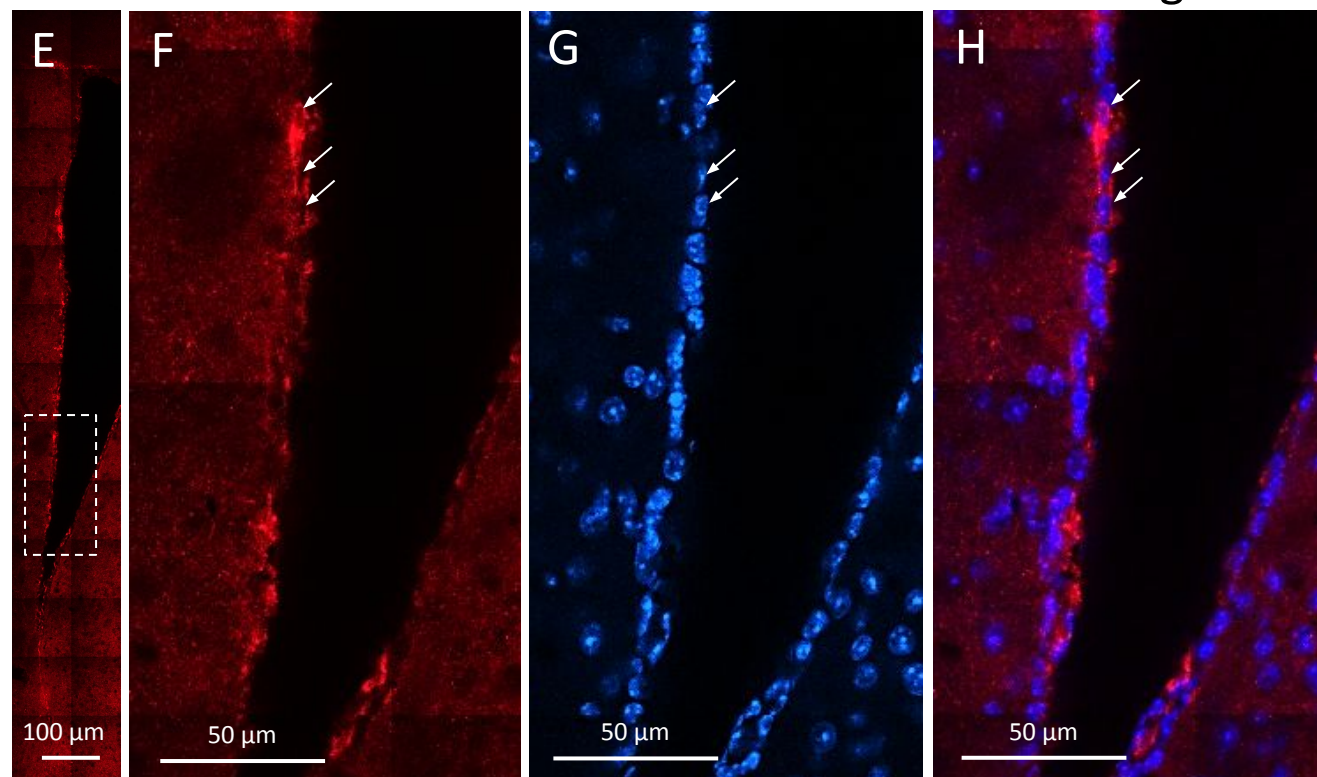

Supplement: Supplementary file 1 [file cells-11-00135-s001.zip › cells-1467224_Suppl. Fig.S1_EGFR in SVZ.pdf]

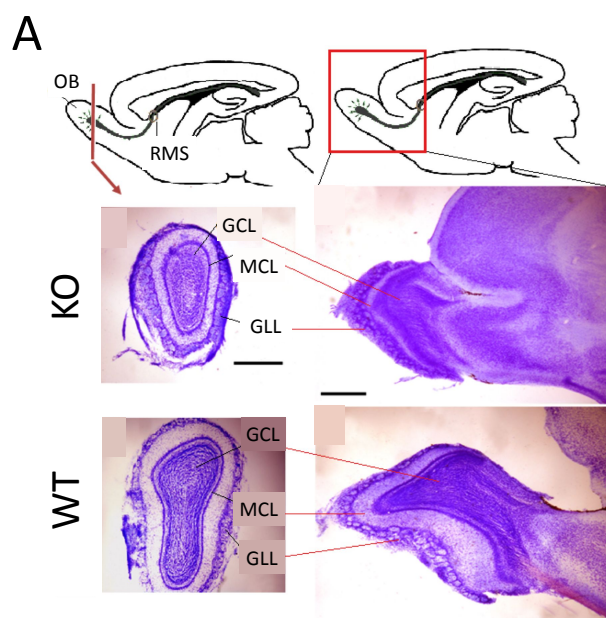

Supplement: Supplementary file 1 [file cells-11-00135-s001.zip › cells-1467224_Suppl. Fig.S2_Nissl.pdf]

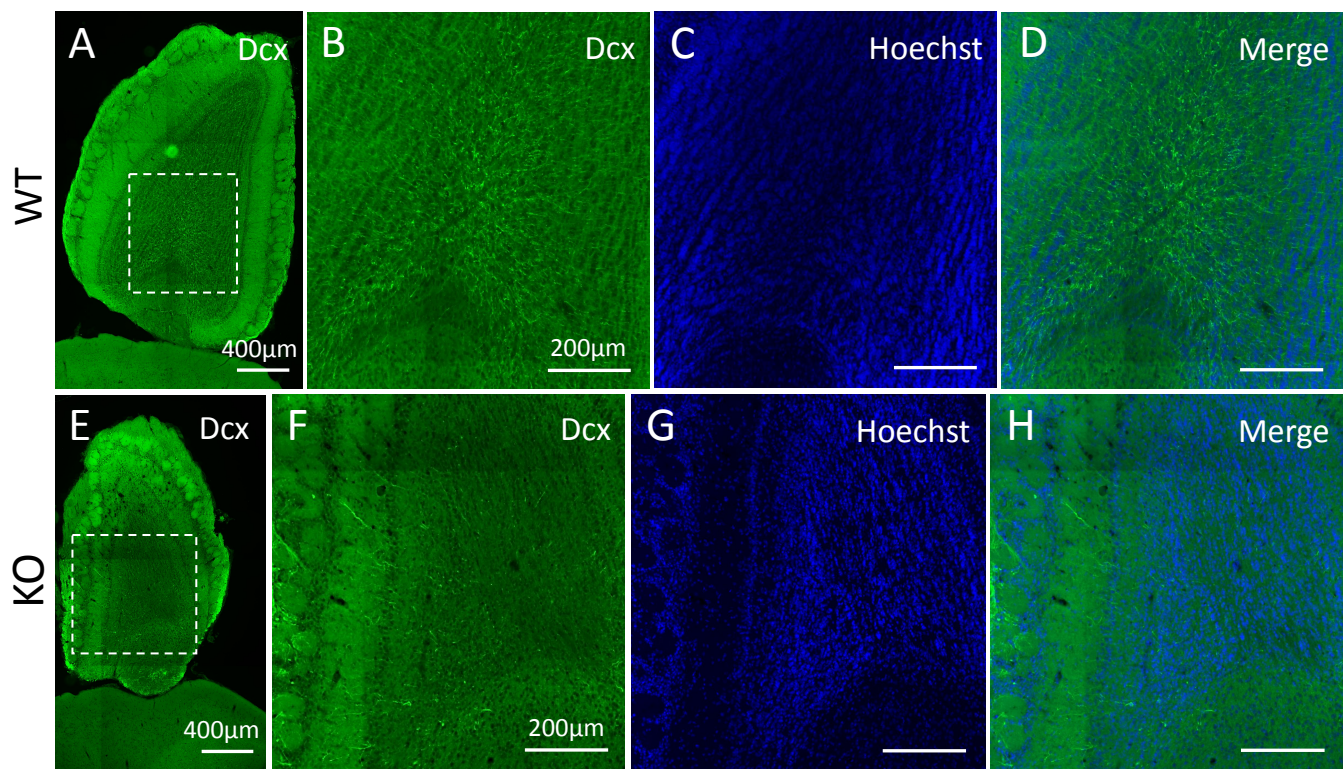

Supplement: Supplementary file 1 [file cells-11-00135-s001.zip › cells-1467224_Suppl. Fig.S3_Dcx in OB.pdf]

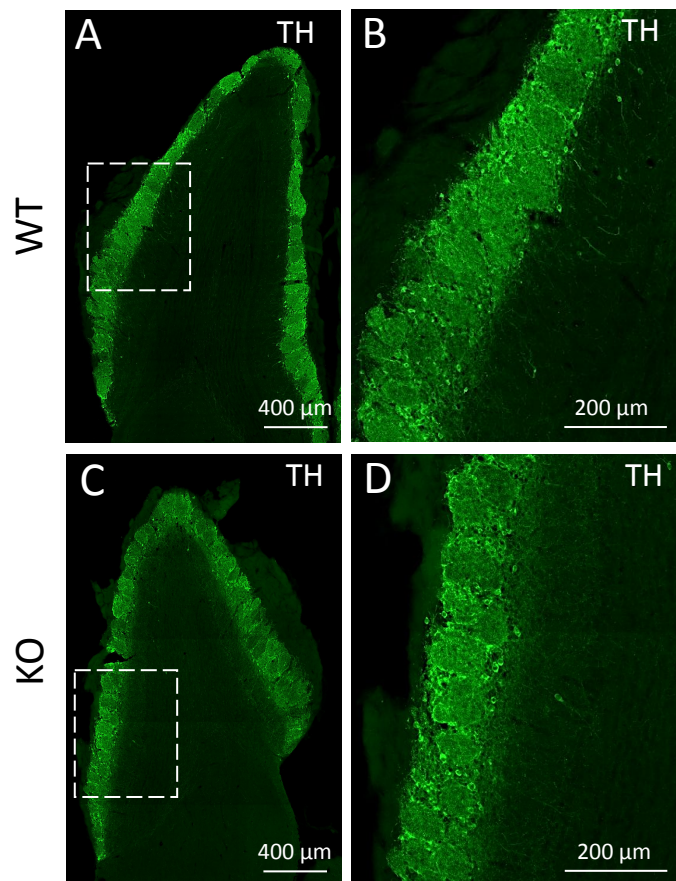

Supplement: Supplementary file 1 [file cells-11-00135-s001.zip › cells-1467224_Suppl. Fig.S4_TH in OB.pdf]
